# Scarcity by design: famine, structural violence, and the biosocial inheritance of political deprivation

**DOI:** 10.3389/fpubh.2026.1819688

**Published:** 2026-05-14

**Authors:** Adan Eftekhari, Amira Aker, Khartik Uppalapati, Bram Wispelwey

**Affiliations:** 1 Harvard College, Cambridge, MA, United States; 2 Department of Stem Cell and Regenerative Biology, Harvard University, Cambridge, MA, United States; 3 RareGen Youth Network, Washington, DC, United States; 4 Boston University School of Public Health, Boston, MA, United States; 5 Harvard Medical School, Boston, MA, United States; 6 FXB Center for Health and Human Rights, Harvard University, Cambridge, MA, United States; 7 Division of Global Health Equity, Brigham and Women's Hospital, Boston, MA, United States

**Keywords:** Colonialism, developmental origins of health and disease, famine, health disparities, health equity, social medicine, state-sanctioned violence, structural violence

## Abstract

Famine is conventionally understood as a crisis of food supply, yet historical and contemporary evidence reveals it as a product of structural violence: political decisions, colonial extraction, and military force that manufacture scarcity and inscribe its consequences onto human bodies across generations. This conceptual analysis examines the intergenerational biology of famine through three cases: the 1944–45 Dutch Hunger Winter, colonial-era famines in India, and the ongoing famine in Gaza. Drawing on the developmental origins of health and disease (DOHaD) paradigm, social medicine, and global health scholarship, we argue that famine's metabolic, immunological, and neurodevelopmental consequences are enabled not only by the severity and timing of caloric deprivation but by the political structures that determine whether deprivation is temporary or sustained, and whether recovery is supported or obstructed. The Dutch case affirms potent within-generation effects partially buffered by postwar welfare reconstruction; colonial India demonstrates how centuries of imperial extraction produced enduring, population-level metabolic vulnerability; and Gaza combines acute starvation with decades of siege, occupation, and infrastructural destruction. A biosocial framework resituates famine as both immediate violence and long-term biological imprinting, urging accountability beyond humanitarian logics of survival to encompass reparative justice, self-determination, and the dismantling of the political systems that produce manufactured hunger.

## 1 Introduction

In Gaza today, parents are skipping meals regularly to save their children meager scraps–if anything at all. A population-based survey conducted in August 2025 found that over 90 percent of households face severe food insecurity, while acute malnutrition among young children has surpassed the thresholds used to declare famine (1–3). Bombardment has destroyed farms, bakeries, water systems, and health facilities; humanitarian convoys wait at sealed borders as children's limbs are measured for wasting in overcrowded shelters. Pregnant women give birth without adequate nutrition, anesthesia, or sterile conditions. Infants who survive delivery face diluted formula, contaminated water, and the immunological vulnerability that comes with being born into a body already marked by deprivation. The sounds of bombardment accompany breastfeeding; displacement disrupts the physiological conditions–caloric intake, hydration, hormonal stability–on which lactation depends (1, 4). This is not an unavoidable shortage but a famine produced by political decisions, military force, and the deliberate dismantling of systems required for life–mass starvation wielded as an intentional warfare tactic, facilitated by the blockade of food, supplies, and aid, and amounting to what international law recognizes as collective punishment (5). The stakes, moreover, extend far beyond the present day: because famine inscribes itself onto bodies and lineages, starvation is not merely the absence of food but a form of biosocial violence that embeds political conditions into biology itself.

The long-term health consequences of famine, particularly for fetuses and infants, are well documented in historical cases, though the picture is more nuanced than often assumed. Research on the Dutch Hunger Winter shows that individuals exposed to famine in utero exhibit higher rates of Type 2 diabetes, cardiovascular disease, and metabolic dysfunction decades later (6). Even in the absence of low birthweight, starvation during gestation alters gene expression and metabolic pathways in ways consistent with the developmental origins of health and disease (DOHaD). A review of historical cohort data demonstrates that the timing and nature of famine exposure produce strikingly different long-term outcomes, including the possibility that postnatal childhood exposure to food deprivation may, paradoxically, be associated with increased longevity in subsequent generations through autophagy-related epigenetic mechanisms, complicating any simple narrative about famine's biological legacy (7). Yet the effects in the Dutch case appear largely limited to the directly exposed cohort and the first generation (6). This contrasts sharply with India, where contemporary high rates of diabetes stem not from a single famine but multiple generations of chronic deprivation.

Whereas the Dutch Hunger Winter lasted approximately six months, British colonial policies imposed recurring famines and systematic undernutrition across the Indian subcontinent from the late eighteenth century through independence in 1947–nearly two centuries of deprivation encompassing not only food, but the full array of dispossession, exploitation, and social suffering wrought by colonialism, which cautions against overinterpreting the specific role of famine periods relative to other ravages of colonial rule (8, 9). This prolonged undernutrition reduced metabolic capacity across populations, a vulnerability forged within the political economy of British colonialism, where export-oriented agriculture, land dispossession, and enforced cash-crop cultivation restructured food systems in ways that made famine a predictable outcome of imperial governance rather than ecological anomaly (8, 9). Notably, these structural conditions did not dissolve with independence; postcolonial India inherited fragmented food systems, entrenched rural poverty, and health infrastructures withered by decades of extractive neglect, ensuring that metabolic risk is primarily reproduced through ongoing social and political conditions rather than through biological inheritance alone, which may play a contributory but secondary role relative to the chronic social suffering that persists through the lifecourse and across generations.

Gaza today sits at the crossroads of these histories. Under siege, pregnant mothers give birth while severely malnourished, dehydrated, and displaced; infants are fed diluted formula or unsafe water; breastfeeding is hindered by hunger, stress, and lack of privacy (1, 4). Even if famine ends tomorrow, its biological aftermath will not: clinical and epidemiological evidence demonstrates that severe malnutrition weakens cardiac muscle, impairs immune function, disrupts neurodevelopment, and reduces cardiovascular capacity in ways that persist into adulthood and resist reversal by refeeding alone (10–13). Gaza is thus not only a humanitarian crisis in the present; it is a site where the intergenerational biology of famine is unfolding in real time. The question becomes: will Gaza resemble the Dutch case, where effects remain largely within one generation, or the Indian case, where structural and political conditions produce persistent, population-level metabolic risk? The answer hinges less on speculative epigenetic mechanisms and more on whether the political structures producing deprivation–occupation, blockade, siege–are dismantled and replaced by conditions of self-determination, restitution, and sustained social protection, as the Dutch population received in the postwar period.

To make sense of these divergent biological and historical patterns requires a shift away from narrow biomedical interpretations. The same structures that produce famine–occupation, blockade, bombardment, forced displacement, and deliberate deprivation–also produce the metabolic, immunological, and developmental vulnerabilities that will shape the lives of survivors. Social medicine emphasizes precisely this point: illness originates in social conditions before it ever appears in the clinic, and the causes of the causes must be confronted if we are to grasp who becomes sick and why (14). Farmer (15) reinforces the moral stakes of that insight; structural violence, he argues, becomes inscribed on the bodies of the poor when political decisions determine exposure to starvation, infection, and early death. In Gaza, what is often described as a “resource-scarce setting” is in fact the predictable outcome of political choices that manufacture scarcity and then treat it as natural (16) –a depoliticization that extends to global health itself, where expert analyses recast crises like famine as technical failures rather than consequences of power (17).

These dynamics are reinforced when international actors respond to Gaza with lowered expectations or diminished standards of care, perpetuating the colonial thinking that remains ingrained in global health practice (18). To analyze the unfolding famine without situating it in these intertwined biological and political forces would risk reproducing the very conceptual blind spots that have long constrained the field's ability to protect those most harmed by structural violence. This paper therefore aims to examine the intergenerational biology of famine through three comparative cases–the Dutch Hunger Winter, colonial India, and contemporary Gaza, occupied Palestine–in order to demonstrate how political structures determine famine's biological reach, and to argue that a biosocial, decolonial framework is necessary for understanding and confronting the manufactured scarcity that inscribes political violence onto human bodies across generations.

## 2 Famine as biological insult and political project: a biosocial framework

To analyze famine as both a biological insult and a political project requires a framework that can hold molecular processes and power structures within the same analytic space–a project that resonates with Nancy Krieger's ecosocial theory of disease distribution and with the tradition of Latin American Social Medicine, both of which insist on theorizing the social determination of health across multiple levels of organization simultaneously (19). The DOHaD paradigm provides one entry point by affirming that prenatal and early-life environments “program” long-term metabolic trajectories, as the Dutch Famine Birth Cohort confirms (6). The within-generation effects of famine on metabolic and developmental outcomes are well established and not in dispute. What remains far more speculative is the extent to which these alterations transmit across generations through strictly biological mechanisms such as epigenetic inheritance, as opposed to through the persistence of the social and political conditions that produced them.

Evolutionary and ecological approaches further illuminate how bodies carry the imprint of deprivation into new social environments. The “capacity-load” model posits that chronic undernutrition constrains metabolic capacity–limiting muscle mass, β-cell development, and glucose handling abilities–such that when postfamine conditions introduce increased caloric availability or rapid nutritional transitions, metabolic load overwhelms this diminished capacity, predisposing individuals to Type 2 diabetes (8). These biological frameworks, however, demand careful scrutiny: attributing metabolic disease to inherited genetic or epigenetic traits prompted by ancestral scarcity carries a troubled history, one intertwined with scientific racism and the geneticization of health disparities in Indigenous and colonized populations, as examination of the “thrifty gene” hypothesis demonstrates (20). How these biological processes interact with political history differs across cases, as the following sections explore.

Social medicine expands this analysis by insisting that biological outcomes cannot be separated from the structures that produce them. Medicine bears an ethical duty to confront the structural forces–poverty, racism, colonialism, and war–that create the causes of the causes and become embodied as disease (14, 15). Yet American medicine has systematically sidelined this tradition by prioritizing individualized, biologically centered explanations of disease over structural analyses of inequality, narrowing the profession's conceptual means for understanding population-level suffering (21). This marginalization reverberates in global health responses to famine, where interventions continue to target individual bodies rather than the political and economic systems that produce mass deprivation, explaining why famine is so often misframed as a technical failure of food delivery rather than a predictable outcome of political arrangements and historical power. Indeed, the DOHaD paradigm itself is not immune to this depoliticizing tendency. When applied without explicit attention to the social and political conditions that produce early-life adversity, DOHaD research can inadvertently locate the origins of health disparities within individual bodies–in epigenetic marks, metabolic set points, or developmental trajectories–rather than in the structures of power that created those exposures. The biosocial framing advanced here is therefore not merely additive but corrective: it insists that the biological sciences of famine be read through, and remain accountable to, the political conditions that make famine possible.

## 3 Epistemic dimensions of famine governance and global health

The epistemic critiques outlined above–the depoliticization of suffering within global health (17), the interrogation of manufactured scarcity (16), and the identification of colonial logic in humanitarian response (18)–have concrete implications for famine governance. When influential actors frame famine as a logistical challenge rather than an outcome of policy, they justify the very hierarchies that created the crisis.

Perceiving famine through this lens also elucidates why interventions so often fail to challenge the systems that enabled hunger in the first place. Historical analyses of mortality decline demonstrate that medical technologies alone cannot address crises stemming from structural violence; improved survival has always depended on both biomedical advances and political-economic reforms that expand access and dismantle inequity (22). These principles translate into specific policy demands for Gaza: ending the illegal siege and blockade, ensuring unimpeded humanitarian access, and addressing the deep structural causes of forced displacement, land dispossession, and de-development to which Palestinians have been subjected–including the full realization of the right to return, sovereignty, and self-determination (5). Yet famine governance remains fraught with a similar tension: modern food-security institutions sometimes obscure responsibility by embedding famine within technical assessments rather than political accountability (23), while explanatory models emphasizing aggregate food supply can erase the mechanisms of entitlement loss and coercion that define how famine unfolds (24). Even the representation of famine carries epistemic weight: images of starvation circulated without context risk transforming complex violence into consumable spectacle, narrowing public understanding at the very moment deeper structural analysis is most critical (25). This dynamic has been especially pronounced in the case of Gaza, where images of starving children have saturated global media while the political mechanisms producing that starvation–border closures, aid obstruction, agricultural destruction–receive comparatively little sustained attention. The result is a representational economy in which suffering becomes visible but its causes remain obscured, inviting humanitarian sentiment while deflecting political accountability. Together, these critiques underline famine as an embodied outcome of political decisions, epistemic frameworks, and representational practices, all of which dictate not only who suffers today but how that suffering will be carried into future generations.

## 4 The Dutch hunger winter: acute deprivation and postwar recovery

Events such as the Dutch Hunger Winter must be understood as “accessibility constraints” engineered through power, not as natural phenomena (24). Caloric rations in cities such as Amsterdam dropped to 400–800 calories per day, with urban residents, especially pregnant women, bearing the brunt of deprivation. This setting, in which a clearly defined population was exposed to abrupt and severe caloric collapse, created the conditions for what would become the Dutch Famine Birth Cohort, a natural experiment that has allowed researchers to trace famine's biological imprint across the lifespan. That the Dutch case is so readily recognized as a political crime–a Nazi-imposed blockade against a European population–is itself telling; colonial and racial hierarchies make it far more difficult for the international community to perceive the same dynamics of engineered starvation when the victims are colonized, racialized, or Indigenous populations, as in India under British rule or in Gaza under Israeli occupation. The timing of prenatal exposure mattered profoundly: early-gestation exposure produced changes in glucose tolerance, lipid profiles, and cardiovascular risk decades later, even when birth weight was normal, evidencing that the most consequential fetal programming can be metabolically “invisible” at birth (6). Mid- and late-gestation exposures showed different patterns, reinforcing that fetal development is not uniformly sensitive but temporally differentiated.

What followed the famine is equally important for interpreting its biological legacy. Postwar reconstruction in Western Europe, characterized by rising wages, social welfare programs, universalizing health systems, and increasingly effective biomedical interventions, provided many families with structural supports that buffered the long-term harms of early-life deprivation. This context matters: survivors of the Dutch famine aged into a society that, despite its inequalities, invested heavily in public health infrastructure and social protections. As a result, while the Dutch case remains foundational for demonstrating potent within-generation DOHaD effects, its transgenerational metabolic consequences appear comparatively limited and highly context-dependent. The cohort's scars are undeniable, but their children and grandchildren were born into political, economic, and nutritional environments fundamentally different from those that shaped gestational life in 1944–45–an essential distinction when comparing the Dutch case to colonial India and contemporary Gaza, where structural deprivation has been neither temporary nor followed by sustained social and political protection.

## 5 Colonial India: chronic imperial extraction and metabolic vulnerability

The contrast with India illuminates how famine's biological imprint deepens and expands when scarcity is not an acute wartime rupture but a chronic condition produced through decades of imperial extraction. Whereas survivors of the Dutch Hunger Winter emerged into a postwar welfare state, populations in colonial India endured repeated, widescale famines generated through British policies that, from the late eighteenth century through independence in 1947, converted subsistence landscapes into export-oriented economies. Grain price liberalization, aggressive taxation, and enforced cash-crop cultivation undermined local food systems and rendered seasonal variability into catastrophic deprivation, as crises commonly attributed to “shortages” were in fact deliberate denials of access enforced through political and economic power (9, 24). This manufactured deprivation was not episodic but systematic, transforming gestational environments for generations and fundamentally altering how bodies calibrated metabolism to uncertainty. The capacity-load model introduced in Section 2 finds its most extensive historical expression in this context: generations of constrained nutrition reduced the metabolic capacity of successive populations–smaller pancreatic β-cell mass, lower lean muscle reserves, diminished glucose handling–while postcolonial nutritional transitions introduced caloric loads that this constrained physiology was ill-equipped to manage (8).

The landscape of chronic deprivation and rapid postcolonial transition that followed British rule captures the role of developmental plasticity under conditions of sustained socioecological stress. Independence did not produce a clean metabolic break. Postcolonial India inherited health systems shaped by decades of imperial underinvestment, and subsequent development policies often compounded rather than corrected these legacies. Structural adjustment programs imposed during the 1980s and 1990s contracted public health spending at precisely the moment chronic disease burdens were accelerating, while the Green Revolution, though it increased aggregate food production, did so unevenly, favoring commodity grains over diverse, micronutrient-rich diets and deepening inequalities between irrigated and rain-fed regions (8). Today's diabetes epidemic cannot be separated from these histories of famine, inequality, and colonial restructuring of food economies–a “colonial burden” whose weight persists in population-level metabolic vulnerability (9). These vulnerabilities are compounded by contemporary health systems that have justified substandard care and a lack of urgency in addressing health problems for poorer countries, a double standard that ensures metabolic risk is both inherited and institutionally reproduced (18). The throughline is clear: the same political forces that once starved tens of millions now determine who bears the world's heaviest diabetes burden and who receives adequate medical care (15). In India, then, famine's metabolic consequences are not confined to a single exposed cohort but are just one component of an imperial project with consequences distributed across time and class, influencing population-level health in ways that make the case a significant means for understanding the unfolding crisis in Gaza.

## 6 Contemporary Gaza: engineered famine under siege and occupation

This continuity is crucial: the forces that once reshaped India's food landscape through taxation, extraction, and blockade now appear in contemporary form through the destruction of Gaza's agricultural system–part of a broader campaign of dispossession across Palestine–alongside restrictions on aid and the systematic interruption of water, fuel, and medical supplies. These policies have produced extreme food insecurity, with households surviving on fractions of the caloric intake required for basic physiological function (1). The WHO's 2025 famine declaration and the IPC Review Committee's confirmation that mortality and malnutrition thresholds have been surpassed (2, 3) underscore that Gaza is not experiencing a humanitarian “shortfall” but a famine whose mechanisms are traceable and deliberate. It is worth noting a tension here: the IPC classification system, critiqued elsewhere in this analysis as part of the technical apparatus that can depoliticize famine, nonetheless serves an important evidentiary function in confirming what populations on the ground already know. The issue is not the data these instruments produce but the political work they are made to do–whether they are deployed to compel accountability or to substitute technical description for structural intervention. Fields have been bulldozed, fishing zones closed, bakeries bombed, and supply routes sealed (26). The collapse of sanitation and water systems produces an environment where hunger overlaps with toxins, dehydration, and infectious disease, exacerbating both immediate and long-term health risks (4).

Notably, the manufactured food insecurity unfolding in Gaza today did not begin in October 2023. For decades, the blockade has restricted caloric intake, constrained agricultural production, and limited economic development–a sustained history of deprivation whose roots extend to at least 2007 and, as 2008 Israeli government documentation reveals, involved calculating the minimum calories necessary to avert malnutrition as a means of calibrating aid deliveries (27). The post-October 2023 escalation has thus intensified a preexisting structure of deprivation into full-scale famine, demonstrating, as in India under colonial rule, scarcity as something engineered rather than inevitable.

These conditions directly affect how famine becomes biologically embodied, especially for pregnant women, fetuses, and infants. Systematic reviews confirm that severe malnutrition produces lasting cardiac structural changes, including reduced ventricular capacity and elevated vascular resistance, alongside immune suppression, impaired neurodevelopment, and diminished cognitive function that persist well beyond the period of acute deprivation (10–13). These findings align with DOHaD evidence from the Dutch cohort (6), but what distinguishes Gaza is that the underlying political structure producing deprivation is ongoing. Unlike postwar Europe's reconstruction or India's eventual, if uneven, food transitions, the metabolic patterns seen in the Dutch cohort and the capacity-load vulnerabilities documented in India may converge in Gaza under conditions of chronic siege and environmental collapse. The capacity-load framework is again instructive: decades of blockade have already constrained the metabolic capacity of Gaza's population, and the acute caloric collapse since October 2023 has imposed an additional, devastating load onto physiology that was never permitted the conditions for full developmental expression. The Dutch cohort's finding that gestational timing of exposure profoundly shapes long-term outcomes carries particular gravity in this context: under conditions of continuous bombardment, forced displacement, and protracted siege, the timing of prenatal exposure to starvation cannot be predicted, controlled, or confined to a single trimester, meaning that the full spectrum of gestational vulnerability is simultaneously in play across Gaza's pregnant population. Yet without sustained attention to these structural forces, Gaza risks being miscast as a “resource-poor setting” rather than a population forcibly deprived (14), and images of starving children, circulated without context, risk converting political violence into consumable spectacle (25). The images out of Gaza of desperate, starving children permeating our screens despite uneven global media visibility, then, are not only enduring famine–they are carrying its future imprint within a world that continues to normalize the conditions that made it possible.

## 7 Comparative synthesis: political architecture and biological reach

The metabolic legacies to emerge in Gaza mirror elements of both Dutch and Indian cases, but each famine's political architecture configures its biological reach in distinct ways. While the Dutch Hunger Winter was a brief but intense blockade and India's famines were the result of recurring, policy-driven scarcity under colonial rule, Gaza's deprivation stems from protracted siege, militarized enclosure, and systematic destruction of the infrastructures that sustain life. Across these settings, famine is not an accident of insufficient food supply but a consequence of access denied through political power (24). Yet the temporalities differ profoundly: the Dutch famine lasted six months; India's chronic undernutrition spanned over 180 years; Gaza's deprivation is both acute and chronic, embedded in decades of occupation and blockade and punctuated by destructive cycles of war. These political differences dictate who is exposed, for how long, and under what intergenerational conditions.

As such, Gaza combines elements of both historical patterns–acute collapse superimposed on generations of constrained nutrition, restricted movement, and environmental degradation. Israel's sustained policy of restricting caloric intake to Gaza's population has longstanding institutional roots, establishing a framework whose explicit aim was no prosperity, no development, no humanitarian crisis (27). Clinical and epidemiological evidence leaves little doubt that these conditions will leave permanent consequences among today's fetuses and infants, including impaired cardiac development, immune suppression, and neurocognitive deficits (10–13). Whether these changes extend into future generations depends less on autonomous biological processes than on whether the political forces sustaining deprivation persist across time.

Health systems, critically, amplify or attenuate this trajectory: postwar Europe built welfare states and universal medical systems that buffered long-term harm (6), while India's fragmented postcolonial health landscape–marked by inequalities, underinvestment, and global health's acceptance of a lower standard of care (9, 18)–left metabolic risk more widely entrenched. Gaza's health system, repeatedly destroyed and constrained, has little capacity to mitigate either immediate starvation or long-term chronic disease. Together, these comparisons underscore that famine's generational reach expands when deprivation is prolonged, recovery is politically obstructed, and health infrastructures are systematically weakened.

The contrast in interventions across all three cases reveals how political choices decide who receives meaningful protection and medical investment and who is left to bear the biological fallout–Europe rebuilt through structural reforms and robust care, India faced punitive relief and market-induced neglect, and global health systems continue to justify limited treatment in places where scarcity was historically manufactured. In Gaza today, interventions largely exist within humanitarian frameworks: food distributions that cannot reach besieged populations, emergency medical care conducted in collapsing hospitals, and technical assessments such as IPC classifications and WHO declarations that describe the crisis without altering the forces sustaining it. Yet any such intervention is undercut by continuous bombardment, border closures, broken ceasefires, and environmental destruction (4, 26). Nonetheless, interventions across all three paradigms reveal not only differing capacities but differing valuations of human life. Where political structures support reconstruction, metabolic sequelae can be contained; where structural violence persists, famine becomes a multigenerational inheritance.

## 8 Discussion

A biosocial perspective makes evident that famine is never merely about lacking calories–it is a process where political decisions, economic hierarchies, and colonial power take physical form in human bodies. As de Waal (28), one of the leading voices and analysts on famine in war, writes of Gaza: Israel “controls every shekel, every sack of flour, every connection to the outside world,” making the territory a site where we will discover how much nutritional stress a population can withstand. These injuries do not arise on their own; they follow the contours of inequality that determine which groups face chronic deprivation, acute shocks, or protection altogether. The lasting diabetes risk in India, the cellular aftermath in the Dutch cohort, and the metabolic futures expected to unfold for children in Gaza all highlight how power directs whose bodies carry the burden. A biosocial framework, then, does not merely describe famine but demands action, defining it as an emergency that nutrition or medicine alone cannot solve.

This recognition reconfigures what responsibility looks like. If starvation imprints biological harm that transcends generations, then preventing and repairing these harms becomes an ethical obligation that exceeds short-term humanitarian action. In colonial India, where diabetes constitutes a “colonial burden,” reparative justice would require confronting food systems, trade dynamics, and health financing arrangements forged by imperial domination–structures that endure today in unequal agricultural markets and underfunded chronic disease care (9). In Gaza, the siege and blockade that produced famine carry obligations for immediate and permanent ceasefire, unrestricted humanitarian and journalist access, infrastructural reconstruction, environmental remediation, long-term metabolic and mental health support calibrated to the known biological consequences of early-life deprivation, and–crucially–the realization of Palestinian self-determination, restitution, and reparations. Decolonizing famine response therefore requires abandoning the minimalist humanitarian logic that settles for survival rather than wellbeing; accepting lower standards of care for the world's poor is itself a colonial artifact (18). A biosocial, decolonial approach must instead call for policies and narratives that restore political and military accountability, invest in chronic disease care, dismantle structural blockades, and protect the futures of those whose bodies bear the traces of manufactured hunger. Whether global health institutions prove capable of receiving this call, or whether their institutional commitments to state partnerships, donor structures, and technical neutrality render them structurally resistant to it, remains an open and urgent question.

Altogether, the Dutch Hunger Winter, colonial India, and contemporary Gaza reveal famine as an inherited crisis constituted by history, power, and policy. The Dutch case corroborates the classic DOHaD pattern–brief but severe deprivation producing lifelong metabolic risk in the directly exposed cohort, partially buffered by postwar welfare systems and high-standard medical care. Colonial India represents a more far-reaching pattern: repeated, structurally induced scarcity generating population-level vulnerability to diabetes, carried into the present through political economy and ongoing structural violence. Gaza now stands at the intersection of these trajectories, with acute starvation imposed upon decades of settler colonial dispossession, occupation, infrastructural collapse, and environmental destruction. The metabolic and cognitive injuries unfolding in the region will not be limited to this moment; they are likely to impact the health of future generations unless the entities producing deprivation are dismantled. Global health, as a profession and academic discipline, is confronted with a choice: maintain narratives that naturalize scarcity, individualize disease, and justify diminished standards of care, or embrace a biosocial, decolonial social medicine that challenges the political forces determining who starves, who survives, and who is granted the conditions to thrive. Reimagining global health through this lens changes the stakes of intervention: it widens the frame from saving lives today to protecting the futures of those whose physiology is being inscribed by violence. In doing so, it elucidates not only what must be understood–but what must be changed–in order to determine who lives, who dies, and whose futures are safeguarded against the metabolic afterlives of famine.

## Data Availability

The original contributions presented in the study are included in the article, further inquiries can be directed to the corresponding author.
